# Data of *de novo* genome assembly of the *Chlamydia psittaci* strain isolated from the livestock in Volga Region, Russian Federation

**DOI:** 10.1016/j.dib.2020.105190

**Published:** 2020-01-27

**Authors:** Valentina A. Feodorova, Sergey S. Zaitsev, Mariya A. Khizhnyakova, Yury V. Saltykov, Vitaliy V. Evstifeev, Fidail M. Khusainov, Sergey I. Yakovlev, Olga S. Larionova, Vladimir L. Motin

**Affiliations:** a Laboratory for Molecular Biology and NanoBiotechnology, Federal Research Center for Virology and Microbiology (FRCViM), Branch in Saratov, 410028, Saratov, Russia; b Laboratory of Viral and Chlamydial Infections, Federal Center for Toxicological, Radiation and Biological Safety, 420074, Kazan, Republic of Tatarstan, Russia; c Department for Microbiology, Biotechnology and Chemistry, Saratov State Agrarian University, 410003, Saratov, Russia; d Department of Pathology, Department of Microbiology & Immunology, University of Texas Medical Branch (UTMB), Galveston, TX 77555, USA

**Keywords:** *Chlamydia psittaci*, Genome assembly, *de novo* genome, Rostinovo-70, Illumina HiSeq 2500 platform, Oxford Nanopore MinION

## Abstract

*Chlamydiae* are obligate intracellular bacteria globally widespread across humans, wildlife, and domesticated animals. *Chlamydia psittaci* is a primarily zoonotic pathogen with multiple hosts, which can be transmitted to humans, resulting in psittacosis or ornithosis. Since this pathogen is a well-recognized threat to human and animal health, it is critical to unravel in detail the genetic make-up of this microorganism. Though many genomes of *C. psittaci* have been studied to date, little is known about the variants of chlamydial organisms causing infection in Russian livestock. This research is the first *de novo* genome assembly of the *C. psittaci* strain Rostinovo-70 of zoonotic origin that was isolated in Russian Federation. The results were obtained by using standard protocols of sequencing with the Illumina HiSeq 2500 and Oxford Nanopore MinION technology that generated 3.88 GB and 3.08 GB of raw data, respectively. The data obtained are available in NCBI DataBase (GenBank accession numbers are CP041038.1 & CP041039.1). The Multi-Locus Sequence Typing (MLST) showed that the strain Rostinovo-70 together with *C. psittaci* GR9 and C*. psittaci* WS/RT/E30 belong to the sequence type (ST)28 that could be further separated into two different clades. Despite *C. psittaci* Rostinovo-70 and *C. psittaci* GR9 formed a single clade, the latter strain did not contain a cryptic plasmid characteristis to Rostinovo-70. Moreover, the genomes of two strains differed significantly in the cluster of 30 genes that in Rostinovo-70 were closer to *Chlamydia abortus* rather than *C. psittaci*. The alignment of the genomes of *C. psittaci* and *C. abortus* in this area revealed the exact boarders of homologous recombination that occurred between two Chlamydia species. These findings provide evidence for the first time of genetic exchange between closely related Chlamydia species.

**Table:** Specifications Table

Subject	Molecular Biology, Veterinary Science
Specific subject area	Genome sequencing
Type of data	Table Graph Figure
How data were acquired	Illumina HiSeq 2500 platform, Oxford Nanopore MinION
Data format	Raw Filtered
Parameters for data collection	Data obtained by using standard protocols of sequencing with the Illumina HiSeq 2500 and Oxford Nanopore MinION technology. Protocols are available on official websites of the companies. Data processing was performed with the use of bioinformatic tools. A PC equipped with Intel Core i7 and 16 GB RAM was used for *de novo* assembly.
Description of data collection	Total DNA of the *C. psittacci* strain Rostinovo-70 isolated from the livestock in Volga Region, Russian Federation was used in the study. *Chlamydia* bacteria were grown in infected chicken embryo, enriched by gradient density centrifugation followed by DNA extraction with the Qiagen DNeasy Blood & Tissue Kit, and then sequenced on the Illumina HiSeq 2500 platform and Oxford Nanopore MinION. Assembler Unicycler was used for *de novo* hybrid assembly with Oxford Nanopore (2.5 GB, 271,098 total sequences) and Illumina (945 Mb, 1,831,776 total sequences) of the filtered reads. Comparative analysis of the Rostinovo-70 chromosome was performed against the plasmidless *C. psittaci* GR9 (GenBank # CP003791.1) using the Mauve software.
Data source location	Federal Center for Toxicological, Radiation and Biological Safety, Kazan, Republic of Tatarstan, Russia, 55° 49′ 49.5516″ N, 49° 3′ 57.8916″ E; Federal Research Center for Virology and Microbiology, Branch in Saratov, Saratov, Russia, 55° 44′ 34.055″ N, 37° 36′ 55.443″ E
Data accessibility	Repository name: GenBank Data identification number: CP041038.1 Direct URL to data: https://www.ncbi.nlm.nih.gov/nuccore/CP041038.1 Repository name: GenBank Data identification number: CP041039.1 Direct URL to data: https://www.ncbi.nlm.nih.gov/nuccore/CP041039.1
Related research article	Valentina A. Feodorova, Sergey S. Zaitsev, Yury V. Saltykov, Vitaliy V Evstifeev, Fidail M. Khusainov, Sergey I. Yakovlev, Olga S. Larionova, Onega V. Ulyanova, Vladimir L. Motin. The molecular characteristics of *Chlamydia psittaci* strain from cattle isolated in the Southeastern European Region of Russia (Volga Region), FEBS Open Bio. 9 (Suppl. 1) (2019) P-02-016. P. 97. https://doi.org/10.1002/2211-5463.12675.

**Table:** 

**Value of the Data** • This is the first report on the *de novo* genome assembly of the *C. psittaci* Rostinovo-70 bacterial strain of zoonotic origin that was isolated in Russian Federation and now available as a reference strain for molecular epidemiology studies. • The genome may be useful for researchers in the fields of molecular biology and epidemiology who study molecular evolution of *Chlamydia* and other intracellular microorganisms with a limited genetic polymorphism. The data obtained may help to complement the large volume of genome level assemblies and should contribute to exploration of microbial taxonomy and evolution. • These data contribute to understanding and improving our knowledge in bacterial diversity and distribution worldwide.

## 1 Data

**Fig. 1:**
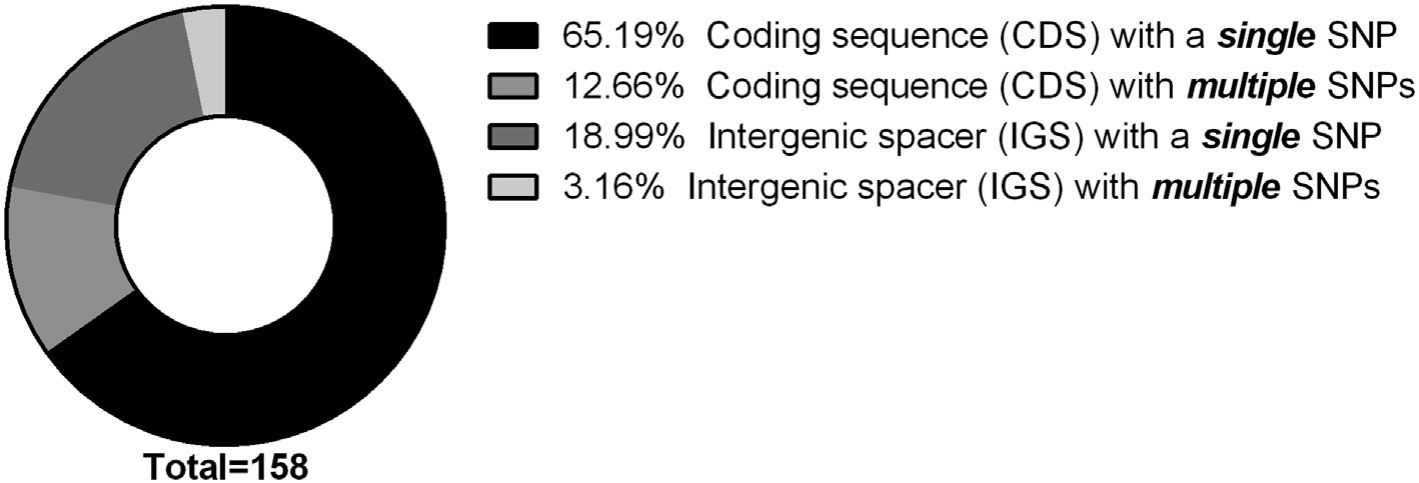
Distribution of all SNPs identified in *C. psittaci* Rostinovo-70 versus *C. psittaci* GR9 strains.

**Fig. 2:**
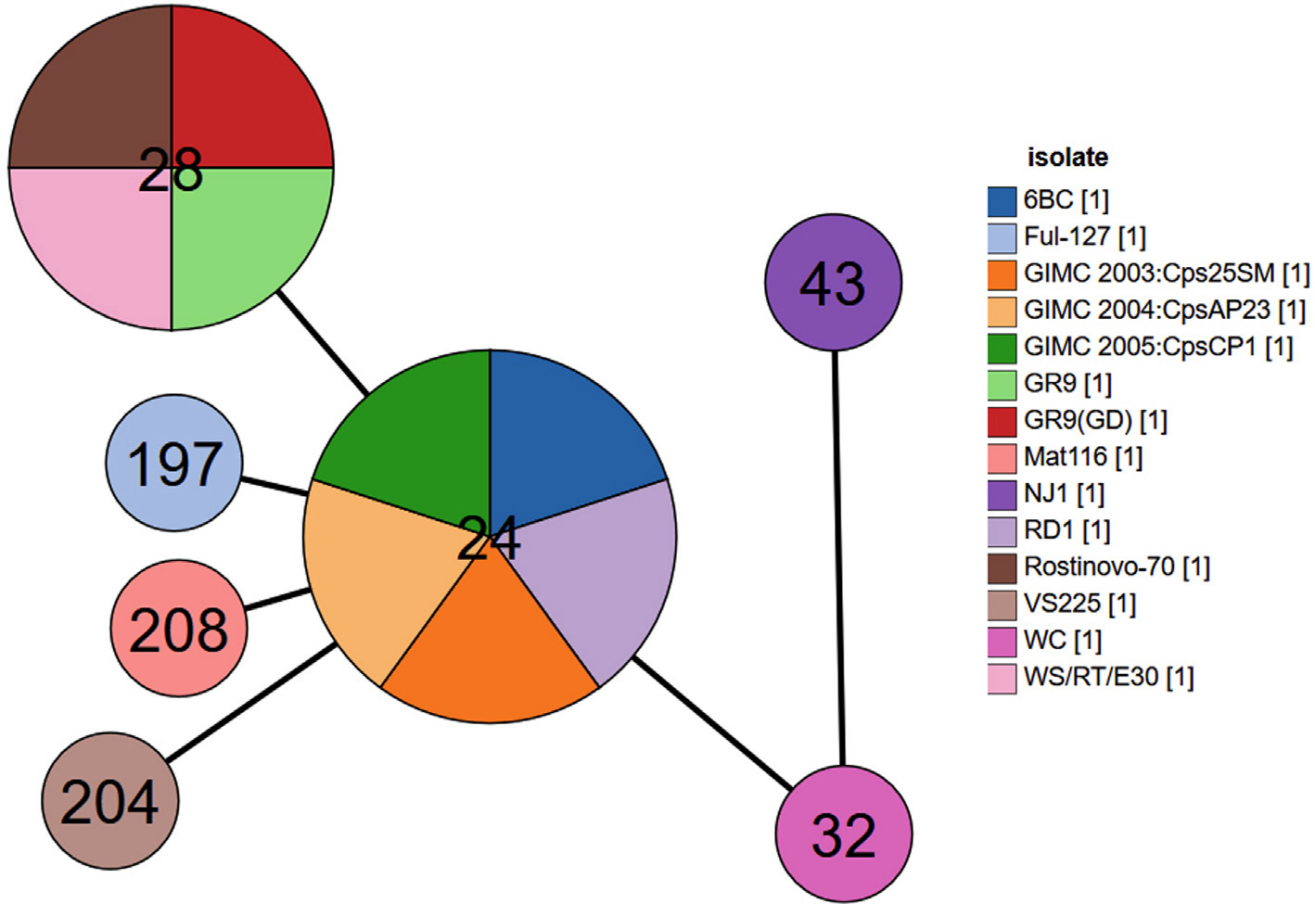
GrapeTree view showing the MLST phylogenetic relationships among *C. psittaci* strains calculated based on the concatenated sequence diversity of seven housekeeping genes (*gatA*, *oppA*, *hfiX*, *gitA*, *enoA*, *hemN*, and *fumC*). The ST28 circle consists of four strains such as *C. psittaci* WS/RT/E30, *C. psittaci* GR9, *C. psittaci* GR9(GD), and *C. psittaci* Rostinovo-70 sequenced in this study.

**Fig. 3:**
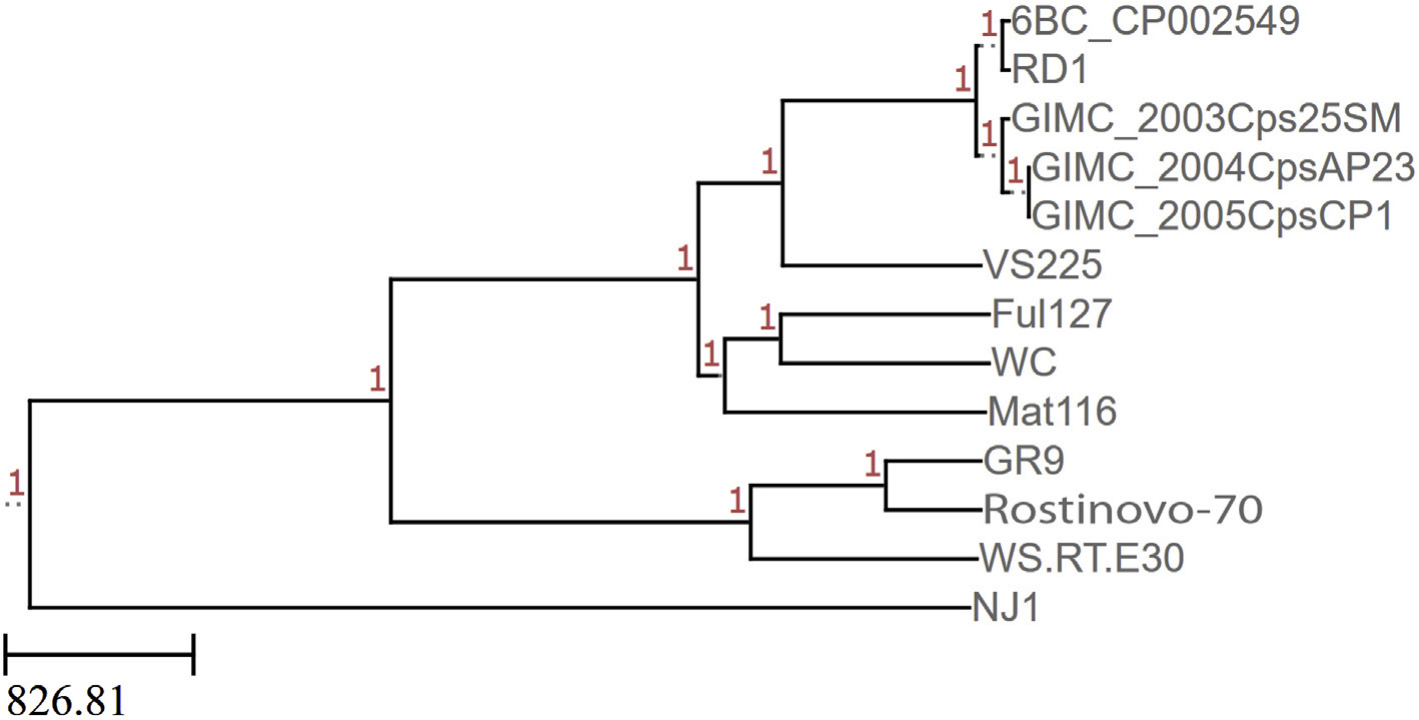
Whole-genome multiple sequence alignments of 12 *C. psittaci* references strains and *C. psittaci* Rostinovo-70 generated into phylogenetic tree calculated using The Reference sequence Alignment based Phylogeny Builder (REALPHY) 1.12 online service, as described in the text.

In this study, we report for the first time a complete genome assembly for the *C. psittaci* wild-type strain Rostinovo-70 sequenced by both the Illumina HiSeq 2500 and Oxford Nanopore MinION platforms. Fig. 1 describes a notable polymorphism with a number of single and multiple single nucleotide polymorphisms (SNPs) in both the coding sequences (CDS) and intergenic spaces in comparison between the *C. psittaci* Rostinovo-70 and the reference genome of *C. psittaci* GR9 strain, isolated from wild ducks in Germany [1]. Fig. 2 demonstrates the phylogenetic structure of 12 homologous reference *C. psittaci* strains and *C. psittaci* Rostinovo-70 strain, which was constructed and visualized by NDtree 1.2 and phylogenetic tree newick viewer, respectively. Fig. 3 demonstrates a phylogenetical separation of the *C. psittaci* Rostinovo-70 and reference *C. psittaci* WS/RT/E30 into two different clades while *C. psittaci* Rostinovo-70 and *C. psittaci* GR9 formed a single clade. Table 1 provides a summary of genome statistical characteristics for the hybrid assembly of the *C. psittaci* Rostinovo-70 by QUAST. Table 2 lists the bioinformatic tools used to analyze the genome of *C. psittaci* Rostinovo-70 strain. Table 3 describes the list of the whole genome *C. psittaci* strains and plasmids used for comparative analysis. Table 4 demonstrates a marked difference in 50 genes between the *C. psittaci* Rostinovo-70 and *C. psittaci* GR9 and the presence of a cluster of 30 genes in the *C. psittaci* Rostinovo-70 that were homologous to *Chlamydia abortus* rather than *C. psittaci*.

**Table 1:** Genome statistical characteristics for the hybrid assembly of the *C. psittaci* Rostinovo-70 by QUAST.

Summary	Assembling results
contigs	2
contigs (≥ 5000 bp)	2
contigs (≥ 10,000 bp)	1
contigs (≥ 25,000 bp)	1
contigs (≥ 50,000 bp)	1
Largest contig (bp)	1,152,559
Total length (bp)	1,160,112
N50 (bp)	1,152,559
N75 (bp)	1,152,559
L50 (bp)	1
L75 (bp)	1
GC (%)	39.08

**Table 2:** The bioinformatic tools used to analyze the genome of *C. psittaci* Rostinovo-70 strain.

Software/Program	Website	Reference
Metagenomics Analysis Server MG-RUST	https://www.mg-rast.org/	[3]
FASTQCv0.11.8	https://www.bioinformatics.babraham.ac.uk/projects/fastqc/	[4]
AfterQC	https://github.com/OpenGene/AfterQC	[5]
Porechop	https://github.com/rrwick/Porechop	[6]
Filtlong	https://github.com/rrwick/Filtlong	[7]
Bowtie2 v. 2.3.5.1	http://bowtie-bio.sourceforge.net/bowtie2/index.shtml	[8]
QUAST	http://quast.bioinf.spbau.ru	[9]
Unicycler	https://github.com/rrwick/Unicycler	[10]
Mauve v. 2.4.0	http://darlinglab.org/mauve/download.html	[11]

**Table 3:** The list of the whole genome *C. psittaci* strains and plasmids used in this study.

Species	Strain	GenBank No.	Reference
*C. psittaci*	Rostinovo-70 chromosome	CP041038.1	This study
	Rostinovo-70 cryptic plasmid	CP041039.1	This study
	GR9 chromosome	CP003791.1	[1]
	CP3 plasmid pcp CP3	CP003813.1	Unpublished
	Rostinovo-70 *omp1*	DQ177459.1	[2]
	Rostinovo-70 *omp2*	DQ177460.1	[2]
	Rostinovo-70 *16S rRNA*	DQ663788.1	[2]
	Rostinovo-70 *23S rRNA*	DQ663789.1	[2]
	Rostinovo-70 plasmid pCp hypothetical protein genes	DQ663790.1	[2]
	WS/RT/E30 chromosome	NC_018622.1	Unpublished
	6BC chromosome	CP002549.1	[12,13]
	RD1 chromosome	FQ482149.1	[14]
	GIMC 2003:Cps25SM chromosome	NZ_CP024453.1	Unpublished
	GIMC 2004:CpsAP23 chromosome	NZ_CP024455.1	Unpublished
	GIMC 2005:CpsCP1 chromosome	NZ_CP024451.1	Unpublished
	VS225 chromosome	NC_018621.1	[1]
	Ful127 chromosome	NZ_CP033059.1	Unpublished
	WC chromosome	NC_018624.1	[1]
	Mat116 chromosome	CP002744.1	Unpublished
	WS/RT/E30 chromosome	NC_018622.1	[1]
	NJ1	CP003798.1	[1]

**Table 4:** Gene polymorphisms between the *C. psittaci* Rostinovo-70 and the reference strains *C. psittaci* GR9 and *C. abortus* strains.

SNPs group	Species & Strain	GenBank No.	Product	Position reference strain	Locus tag reference strain	Locus tag Rostinovo-70	Identity,%
1	*C. psittaci* GR9	CP003791.1	DnaK DNA-3-methyladenine glycosylase family protein	253,092..253,664	B598_0269	FI836_03950	95.29
2			vacB and RNase II 3′-5′ exoribonucleases family protein	253,664..255,709	B598_0270	FI836_03955	93.40
3			chaperone protein	255,866..257,845	B598_0271	FI836_03960	95.30
4			grpE family protein	257,871..258,446	B598_0272	FI836_03965	93.40
5			heat-inducible transcription repressor HrcA	258,443..259,603	B598_0273	FI836_03970	93.36
6			proS prolyl-tRNA synthetase	259,712..261,445	B598_0274	FI836_03975	91.82
7			hypothetical protein	261,710..262,906	B598_0275	FI836_03980	86.80
8			putative lipoprotein	263,013..263,957	B598_0276	FI836_03985	92.28
9			hypothetical protein	263,962..264,240	B598_0277	FI836_03990	92.45
10			ABC transporter substrate binding family protein	263,962..264,240	B598_0278	FI836_03995	93.77
11			ll-diaminopimelate aminotransferase	265,277..266,473	B598_0279	FI836_04000	91.31
12			hypothetical protein	266,738..267,508	B598_0280	FI836_04005	84.77
13			hypothetical protein	267,942..269,177	B598_0281	FI836_04010	92.79
14			hypothetical protein	269,178..271,262	B598_0282	FI836_04015	92.82
15			hypothetical protein	271,402..272,007	B598_0283	FI836_04020	96.03
16			hypothetical protein	271,983..272,279	B598_0284	FI836_04025	98.30
17			HIT domain protein	272,276..272,608	B598_0285	FI836_04030	97.00
18			hypothetical protein	272,652..274,268	B598_0286	FI836_04035	92.70
19			hypothetical protein	274,257..274,520	B598_0287	FI836_04040	82.20
20			solute symporter family protein	274,870..276,204	B598_0288	FI836_04045	91.09
21	*C. abortus* LLG	CP018296.1	putative 3-methyladenine DNA glycosylase	253,058..253,630	CAB1_0249	FI836_03950	97.91
22			putative ribonuclease	253,630..255,678	CAB1_0250	FI836_03955	
23			putative 3-methyladenine DNA glycosylase	253,058..253,630	CAB1_0249	FI836_03950	99.12
24			putative ribonuclease	253,630..255,678	CAB1_0250	FI836_03950	
25			heat shock chaperone protein	255,832..257,811	CAB1_0251	FI836_03960	99.29
26			heat shock protein GrpE(hsp-70 cofactor)	257,837..258,412	CAB1_0252	FI836_03965	98.36
27			heat-inducible transcription repressor	258,409..259,569	CAB1_0253	FI836_03970	
28			prolyl-tRNA synthetase	259,678..261,411	CAB1_0254	FI836_03975	98.73
29			hypothetical protein	267,895..269,139	CAB1_0261	FI836_04010	98.95
30			hypothetical protein	271,371..272,240	CAB1_0263	FI836_04025	98.30
31			hypothetical protein	272,614..274,230	CAB1_0265	FI836_04035	98.82
32			hypothetical protein	272,614..274,230	CAB1_0265	FI836_04035	96.59
33			putative sodium symporter	274,832..276,166	CAB1_0267	FI836_04045	96.55
34	*C. abortus* GIMC 2006: CabB577	CP024084.1	heat shock protein GrpE	257,750..258,325	CHAB577_0257	FI836_03965	98.78
35			heat-inducible transcription repressor HrcA	258,322..259,482	CHAB577_0258	FI836_03970	
36			uncharacterized protein	261,590..262,786	CHAB577_0260	FI836_03980	97.16
37			uncharacterized protein	263,842..264,132	CHAB577_0262	FI836_03990	99.43
38			putative ABC transporter substrate-binding protein	264,117..265,160	CHAB577_0263	FI836_03995	
39			l,l-diaminopimelate aminotransferase	265,157..266,353	CHAB577_0264	FI836_04000	
40			putative ABC transporter substrate-binding protein	264,117..265,160	CHAB577_0263	FI836_03995	99.33
41			putative ABC transporter substrate-binding protein	265,157..266,353	CHAB577_0264	FI836_04000	
42			uncharacterized protein	266,316..266,459	CHAB577_0265	FI836_04000	
43			uncharacterized protein	266,618..267,382	CHAB577_0266	FI836_04005	97.64
44			Rossmann fold domain-containing protein	269,053..271,137	CHAB577_0268	FI836_04015	98.94
45			uncharacterized protein	271,285..272,154	CHAB577_0269	FI836_04020	99.01
46			uncharacterized protein	271,285..272,154	CHAB577_0269	FI836_04020	97.00
47			is(5′-nucleosyl)-tetraphosphatase	272,151..272,483	CHAB577_0270	FI836_04030	
48	*C. abortus* GN6	CP021996.1	hypothetical protein	262,872..263,816	CEF07_01315	FI836_03985	99.68
49			hypothetical protein	263,821..264,111	CEF07_01320	FI836_03990	98.92
50			ABC transporter substrate-binding protein	264,096..265,139	CEF07_01325	FI836_03995	

## 2 Experimental design, materials, and methods

### 2.1 DNA extraction, Illumina and nanopore sequencing, and assembly

Total DNA was extracted from the lyophilized chicken embryo tissue that was infected with *C. psittaci* strain Rostinovo-70 followed by density gradient centrifugation. For this purpose the DNeasy Blood & Tissue Kit (250) QIAGEN (Qiagen, Hilden, Germany) was applied. The final DNA concentration was measured using a spectrophotometer from BioRad (Bio-Rad Laboratories, Redmond, WA, USA). Preparation of the DNA library for sequencing was performed using 1D Genomic DNA by ligation SQK-LSK108 (Oxford Nanopore Technologies, Oxford, UK). DNA end repair and dA-tailing steps was performed using NEB repair modules (New England Biolabs, Ipswich, MA, USA). All clean-up steps of DNA preparation were performed using Agencourt AMPure XP beads (Beckman Coulter Life Sciences, Indianapolis, IN, USA). The final volume of prepared DNA was 75 μl. A FLO-MIN-106 R9.4 Flow cell (Oxford Nanopore Technologies, Oxford, UK) was used to perform sequencing with the MinION and software the MinKNOW. In parallel, the extracted DNA was sequenced with the Illumina HiSeq 2500 platform (Genoanalytica, Moscow, Russia, https://www.genoanalytica.ru/).

The sequencing runs generated a total of 3.88 GB (7,493,423 total sequences) of single-end reads by the Illumina platform in FASTQ format and 3.08 GB (1,24 M reads) by the Oxford Nanopore in fast5 format. After filtering out chicken embryo tissue reads, the *C. psittaci* DNA used for *de novo* hydrid assembly was composed with the clean reads for both Illumina (945 Mb, 1,831,776 total sequences) and Oxford Nanopore (2.5 GB, 271,098 total sequences). Assembly analysis showed an availability of the entire chromosome in a single contig (1,171,768 bp length, the GenBank accession number is CP041038.1). Additionally, the presence of *C. psittaci* cryptic plasmid (7678 bp length) was identified as the extrachromosomal replicon (the GenBank accession number is CP041039.1).

In contrast to the plasmidless *C. psittaci* GR9, a crypric plasmid (7659 bp) was detected in the *C. psittaci* Rostinovo-70. In fact, four SNPs and quadruple-SNP combinations (AGAA→TTCT) were found in the *C. psittaci* Rostinovo-70 cryptic plasmid in comparison with the reference *C. psittaci* CP3 plasmid pcp CP3 (GenBank Accession number CP003813.1). The consecutive comparative analysis of several target genes of the *C. psittaci* Rostinovo-70 strain after Sanger sequencing by another group [2], namely the *omp1*, *omp2*, *16S rRNA*, *23S rRNA* and plasmid pCp putative genes (GenBank Accession numbers DQ177459.1, DQ177460.1, DQ663788.1, DQ663789.1 and DQ663790.1, respectively), with the relevant genes of the whole genome sequence of the Rostinovo-70 strain deposited by us demonstrated their complete identity (100%). The only exception was *omp2* (GenBank Accession number DQ177460.1), which showed an identity of 99.83% due to the SNP at position 534 displayed a T→A substitution.

### 2.2 Program and scripts for bioinformatics

Briefly, taxonomic analysis of the raw reads was performed by Metagenomics Analysis Server MG-RUST [3]. Quality assessment of the reads was performed using FASTQCv0.11.8 [4]. Removal of low-quality reads with ambiguous base (N) and the adapter sequences from the Illumina data was made by AfterQC [5]. The Porechop [6] was used to find and remove adapters from Oxford Nanopore reads. The Filtlong software [7] was used to filter short Nanopore reads smaller than 2000 bp. Single-end Illumina reads were filtered using Bowtie2 v. 2.3.5.1 [8]. The reference strains mapping was performed by Bowtie2 v. 2.3.5.1. with 20 reference *C. psittaci* genomes (Table 3) and five *C. psittaci* plasmids deposited in GenBank, which had more than 95% homology to Rostinovo-70. Genome statistical data analysis of the hybrid assembly of the *C. psittaci* Rostinovo-70 was generated with Quality Assessment Tool for Genome Assemblies (QUAST) [9]. Hybrid *de novo* assembly was carried out by using Unicycler assembly pipeline for bacterial genomes [10]. A search of local changes, such as nucleotide substitutions in individual genes, alignment, as well as comparison with the reference genomes were performed by software Mauve v. 2.4.0. [11] allowing more accurate determination of the positions of mutations in coding and non-coding regions.

### 2.3 Phylogenetic analysis

The MLST based on the concatenated sequences of seven housekeeping genes with the use of a DataBase hosted at http://pubmlst.org/chlamydiales/ assigned the *C. psittaci* Rostinovo-70 to sequence type (ST)28. In fact, *C. psittaci* Rostinovo-70, *C. psittaci* GR9, and C*. psittaci* WS/RT/E30 belong to the same ST28 indicating their origination from a single progenitor. Nevertheless, the strains *C. psittaci* Rostinovo-70 and *C. psittaci* WS/RT/E30 (GenBank Accession number NC_018622.1) were separated phylogenetically into two different clades (Fig. 3). In contrast, *C. psittaci* Rostinovo-70 and *C. psittaci* GR9 formed a single clade, despite that they demonstrated a marked difference in 50 genes (Table 4). Further analysis revealed the presence of a cluster of 30 genes that were closer to *C. abortus* rather than *C. psittaci* (Table 4). The alignment of the genomes of *C. psittaci* Rostinovo-70, *C. psittaci* GR9, and *C. abortus* LLG in this area determined the exact boarders of the homologous recombination that occurred between two Chlamydia species, such as *C. psittaci* and *C. abortus*. One region of recombination was located within the gene encoding putative 3-methyladenine DNA glycosylase resulting in the frameshift within the FI836_03950 in Rostinovo-70. The consequence of the alteration of this gene to pseudogene on virulence of this strain will be part of a future investigation. Another region of recombination was localized within the FI836_04045 encoding putative sodium symporter family protein resulting in formation of a hybrid protein between two Chlamydia species. Overall, the comparative genomics appears to reveal the first evidence of homologous recombination between two organisms.
